# Identification of the Maize *PP2C* Gene Family and Functional Studies on the Role of *ZmPP2C15* in Drought Tolerance

**DOI:** 10.3390/plants13030340

**Published:** 2024-01-23

**Authors:** Yunyun Pang, Liru Cao, Feiyu Ye, Chenchen Ma, Xiaohan Liang, Yinghui Song, Xiaomin Lu

**Affiliations:** 1 Grain Crops Research Institute, Henan Academy of Agricultural Sciences, Postgraduate T&R Base of Zhengzhou University, Zhengzhou 450002, China; 18237149973@163.com (Y.P.); caoliru008@126.com (L.C.); 18838971380@163.com (F.Y.); mchenchen1017@163.com (C.M.); yinyutianqi2020@163.com (X.L.); yinghui820@163.com (Y.S.); 2 School of Agricultural Sciences, Zhengzhou University, Zhengzhou 450002, China; 3 The Shennong Laboratory, Zhengzhou 450002, China

**Keywords:** maize, protein phosphatase, drought tolerance, subcellular localization, interacting proteins

## Abstract

The protein phosphatase PP2C plays an important role in plant responses to stress. Therefore, the identification of maize *PP2C* genes that respond to drought stress is particularly important for the improvement and creation of new drought-resistant assortments of maize. In this study, we identified 102 *ZmPP2C* genes in maize at the genome-wide level. We analyzed the physicochemical properties of 102 *ZmPP2Cs* and constructed a phylogenetic tree with *Arabidopsis*. By analyzing the gene structure, conserved protein motifs, and synteny, the *ZmPP2Cs* were found to be strongly conserved during evolution. Sixteen core genes involved in drought stress and rewatering were screened using gene co-expression network mapping and expression profiling. The qRT-PCR results showed 16 genes were induced by abscisic acid (ABA), drought, and NaCl treatments. Notably, *ZmPP2C15* exhibited a substantial expression difference. Through genetic transformation, we overexpressed *ZmPP2C15* and generated the CRISPR/Cas9 knockout maize mutant *zmpp2c15*. Overexpressing *ZmPP2C15* in *Arabidopsis* under drought stress enhanced growth and survival compared with WT plants. The leaves exhibited heightened superoxide dismutase (SOD), peroxidase (POD), ascorbate peroxidase (APX), and catalase (CAT) activities, elevated proline (Pro) content, and reduced malondialdehyde (MDA) content. Conversely, *zmpp2c15* mutant plants displayed severe leaf dryness, curling, and wilting under drought stress. Their leaf activities of SOD, POD, APX, and CAT were lower than those in B104, while MDA was higher. This suggests that *ZmPP2C15* positively regulates drought tolerance in maize by affecting the antioxidant enzyme activity and osmoregulatory substance content. Subcellular localization revealed that *ZmPP2C15* was localized in the nucleus and cytoplasm. Yeast two-hybrid (Y2H) and bimolecular fluorescence complementation (BiFC) experiments demonstrated *ZmPP2C15*’s interaction with ZmWIN1, ZmADT2, ZmsodC, Zmcab, and ZmLHC2. These findings establish a foundation for understanding maize *PP2C* gene functions, offering genetic resources and insights for molecular design breeding for drought tolerance.

## 1. Introduction

Maize (*Zea mays* L.) is widely grown worldwide as a major food crop [1]. In recent years, climate warming and frequent extreme weather events, such as high temperatures and droughts, have significantly impacted corn yield and quality. Drought has become one of the most important abiotic stress factors limiting maize growth, development, and yield [2]. Droughts have led to a number of natural disasters in terms of reduced crop yields [3]. Data show that for maize, drought causes global economic losses averaging between USD 1.5 and USD 20 billion [4]. Therefore, mining the key drought tolerance genes of maize and analyzing their genetic mechanisms provides a theoretical basis for breeding new drought-resistant maize varieties. Ensuring a high and stable corn yield is also of great practical significance.

The protein phosphatase PP2C, the largest family of protein phosphatases in higher plants, is regulated by the dephosphorylation of amino acid residues. Serine and threonine protein phosphatases were the first protein phosphatases identified in plants. Subsequently, genes encoding protein phosphatases were cloned into *Arabidopsis thaliana* [5], tobacco [6], rice [7], wheat [8], and maize [9]. An increasing number of studies have demonstrated that PP2C protein phosphatases are involved in plant growth and development and in a variety of signaling pathways, including abscisic acid (ABA) signaling, abiotic stress response, cell division, and plant immunity. For example, the expression of *TaPP2C-A10* is negatively correlated with plant drought tolerance. Transgenic plants exhibit weak drought tolerance at the seedling stage, while gene knockout markedly enhances drought tolerance [10]. *AtPP2CF1* overexpression in *A. thaliana* accelerates inflorescence stem growth by activating cell proliferation and expansion, resulting in plants with a higher biomass yield [11]. Overexpression of tiger nut *CePP2C19* enhances tolerance in transgenic *A. thaliana* [12]. *GhDRP1* is involved in the response to drought stress by regulating ABA signaling and flavonoid biosynthesis pathways, and *GhDRP1* overexpression plays a negative regulatory role in the response to drought stress in cotton [13]. *ZmPP2C26* negatively regulates drought stress response and photosynthesis by dephosphorylating *ZmMAPK3* and *ZmMAPK7* [14]. Finally, *OsPP65* regulates the rice response to abiotic stress by independently modulating the ABA and jasmonic acid signaling pathways. Moreover, *OsPP65* knockdown enhances rice tolerance to osmotic and salt stresses [15]. These studies suggest that *PP2C* genes enhance plant resistance to abiotic stressors.

Currently, most studies have reported the functions and regulatory mechanisms of *PP2C* genes in the A subfamily. However, gene functions in other subfamilies of *PP2C* have been rarely reported. In this study, 102 *ZmPP2C* genes were identified in the maize genome. We analyzed maize seedling drought-rewatering transcriptome results using gene co-expression networks and expression heat profiling to identify the core node—the PP2C protein phosphatase gene. Using qRT-PCR, we scrutinized the expression of core genes in various tissues and their patterns under drought, NaCl, and ABA treatments. We investigated the drought tolerance of the *ZmPP2C15* gene through genetic transformations. We also characterized the reciprocal proteins of *ZmPP2C15*. These findings lay the foundation for elucidating the functions of the maize *PP2C* gene family, providing genetic resources and insights for molecular design in drought-resistant breeding, thereby accelerating the development of new drought-resistant maize varieties.

## 2. Results

### 2.1. Identification and Evolutionary Analysis of the Maize PP2C Gene Family

In this study, 102 *PP2C* family genes were identified at the whole-genome level in maize, which were sequentially named *ZmPP2C1*-*ZmPP2C102* based on their location on the chromosome (Supplementary Table S1). The amino acid sequences of the *ZmPP2C* gene family ranged from 233 to 964 amino acids, indicating that the protein sequences of the family members differed significantly. The isoelectric points ranged from 4.54 to 9.88, and there were 76 acidic proteins. The molecular weight ranged from 10.61 to 93.1 kD, with *ZmPP2C7* having the largest molecular weight. Protein stability coefficients ranged from 26.65 to 76.65, with 72.55% for unstable proteins. The hydrophilicity coefficient lay between –1.236 and 0.161, and the fat coefficient ranged from 65.77 to 95.92. Subcellular localization predictions indicated that PP2C proteins are predominantly localized in chloroplasts, cytoplasm, and nuclei, with lesser occurrences in cytoskeletal regions, mitochondria, vesicles, and the plasma membrane.

**Figure 1:**
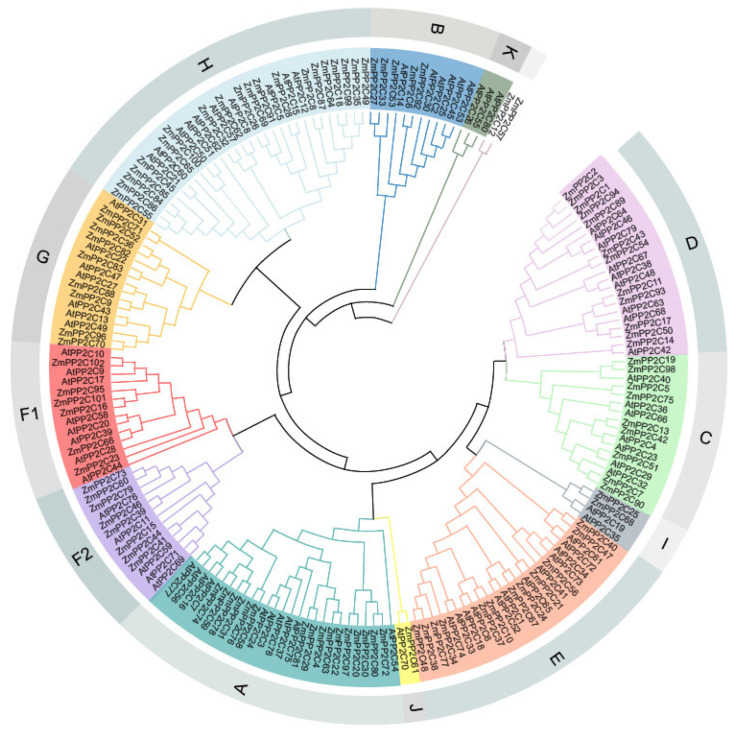
Phylogenetic trees of PP2C family members between *Zea mays* (Zm) and *A. thaliana* (At). A phylogenetic tree was constructed using the neighbor-joining (NJ) method, with 1000 repetitions. Except for ungrouped PP2C proteins, all PP2Cs were classified into 12 subfamilies (A–K), with each subfamily indicated by a different color. The members of the F subfamily are divided into two subfamilies, and the subfamilies are named F1 and F2.

To analyze the evolutionary relationships of maize *PP2C* gene family members, 80 *A. thaliana* PP2C proteins and 102 maize PP2C proteins were used to construct a phylogenetic tree (Figure 1). Moreover, most *PP2C* genes in maize and *A. thaliana* are intermixed in each subfamily. The 102 PP2C proteins were grouped into 12 subfamilies (A–K), whereas ZmPP2C12 and ZmPP2C57 did not cluster with any subfamily. This was similar to the grouping of PP2Cs in *A. thaliana*, or rice. The numbers of *ZmPP2Cs* in subfamilies A and H were 16 and 20, respectively; the number of *ZmPP2Cs* in subfamilies J was 1; and the K subfamily contained no *ZmPP2Cs*. Except for subgroup K, which contained only *AtPP2C* genes, the distributions in the other maize subfamilies were similar to those of *A. thaliana*, suggesting that *ZmPP2Cs* are strongly conserved during evolution.

### 2.2. Distribution of Maize PP2C Family Members on Chromosomes and Analysis of Gene Structure and Protein-Conserved Motifs

The 102 *ZmPP2Cs* genes were unevenly distributed across 10 chromosomes (Supplementary Figure S1). There are 14 genes on chromosome 1, and they form a gene cluster at each end of the chromosome. The end of chromosome 9 forms a gene cluster containing 12 genes. Chromosome 3 has the fewest genes but also forms a small cluster of genes at the end of the chromosome. All other chromosomes contained gene clusters in their telomeres. The results showed that maize *PP2C* genes mostly existed in gene clusters at chromosome telomeres. Genes of the same subfamily did not collocate on the same chromosome, and the number of genes contained in the chromosome did not correlate with chromosome length.

**Figure 2:**
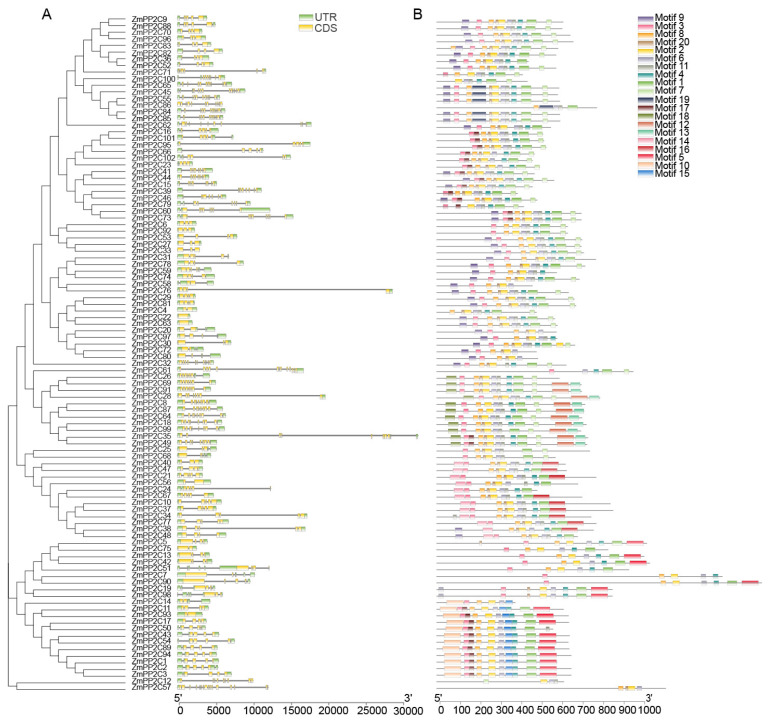
Gene structure of *ZmPP2C* family members. (**A**) Exon intron structure analysis of *ZmPP2C* genes. Exons and introns are represented by orange rectangles and thin stripes, respectively, and untranslated regions (UTRs) are represented by green rectangles. (**B**) Distribution of all ZmPP2C protein motifs recognized by MEME. Different colored rectangles represent different protein motifs.

Analysis of the gene structures of the 102 *ZmPP2Cs* unveiled structural variations (Figure 2A). Those on the same branch of the evolutionary tree exhibited identical or similar gene structures. In maize *PP2Cs*, both the introns and exons cross-set in the complete gene sequence. Among them, *ZmPP2C57* had the highest number of introns, while *ZmPP2C22* and *ZmPP2C63* lacked introns, and *ZmPP2C4*/*56*/*75*/*93* only had one intron. We hypothesized that this might be related to the relative conservation of the *ZmPP2C* gene structure. Furthermore, an examination of the conserved motifs of ZmPP2C proteins (Figure 2B) demonstrated that proteins with close evolutionary relationships shared highly similar motifs. Notably, *ZmPP2C12* and *ZmPP2C57* contained only four motifs, whereas most of the remaining ZmPP2C proteins contained ten motifs, with most featuring the 2/3/4/6/7/8/9/11 motif structures. In addition, the number of motifs in ZmPP2C86 was deficient compared with that in other proteins of the same subfamily, which may be caused by base loss during gene tandem duplication.

### 2.3. Collinearity Analysis between PP2C Gene Families in Sorghum, Rice, Arabidopsis, and Maize

**Figure 3:**
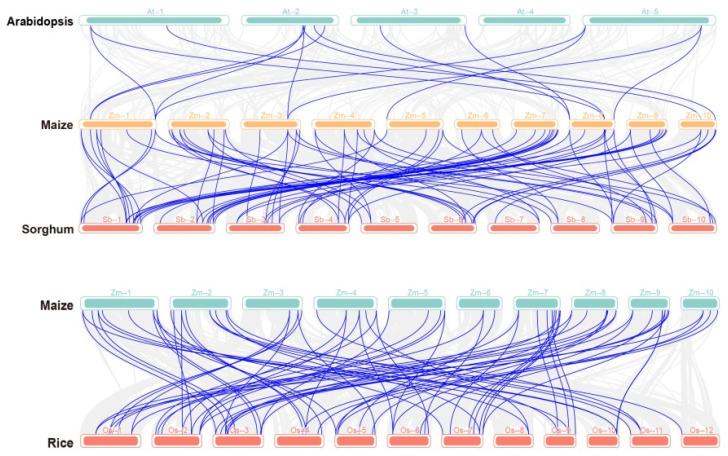
Synteny analysis of *PP2C* family genes in different species. At: *Arabidopsis thaliana*; Os: rice; Sb: sorghum; Zm: maize. The number on the horizontal line for each species represents the chromosome to which it belongs.

To analyze the similarities and differences between maize and the *PP2C* genes of other species, a collinearity analysis was performed for maize, *Arabidopsis*, rice, and sorghum (Figure 3). Replicative relationships were found between maize and *Arabidopsis*, rice, and sorghum, but the collinearity between maize and the dicotyledonous plant *Arabidopsis* was low (only 15 pairs). Several gene pairs exhibited collinearity among maize, rice, and sorghum. Notably, a sorghum/rice *PP2C* gene displayed collinearity with multiple maize *PP2C* genes. Examples include the collinearity between sorghum *SbPP2C35* (*EER90623*) and maize *PP2C13*/*42*, as well as between *SbPP2C46* (*EES17579*) and *ZmPP2C5/13/75*. Additionally, collinearity was identified between rice *OsPP2C15* (*Os02t0567200*) and maize *ZmPP2C18*/*35*/*49*/*99*. These results indicate that *ZmPP2Cs* are strongly conserved during evolution and that the evolutionary process in maize is more complex than that in sorghum and rice.

### 2.4. Analysis of Candidate Genes for ZmPP2Cs under Drought and Rewatering Conditions

Analysis of the expression profiles of *ZmPP2Cs* at different time points using maize transcriptome data and real-time fluorescence quantification revealed that most *ZmPP2Cs* were expressed in both tissues and organs (Supplementary Figures S2 and S3). The expression patterns were significantly different. Among them, *ZmPP2C48* exhibited the highest expression in female spikes, *ZmPP2C44* exhibited the highest expression in male spikes, and *ZmPP2C15/21/29/45/47/58/72* exhibited the highest expression in young roots. *ZmPP2C97* and *ZmPP2C98* showed the highest expression levels in mature leaves, *ZmPP2C89* and *ZmPP2C93* in mature roots, and *ZmPP2C31/35/102* in young leaves. These results suggested that *ZmPP2Cs* functionally diverged during a long evolutionary process.

**Figure 4:**
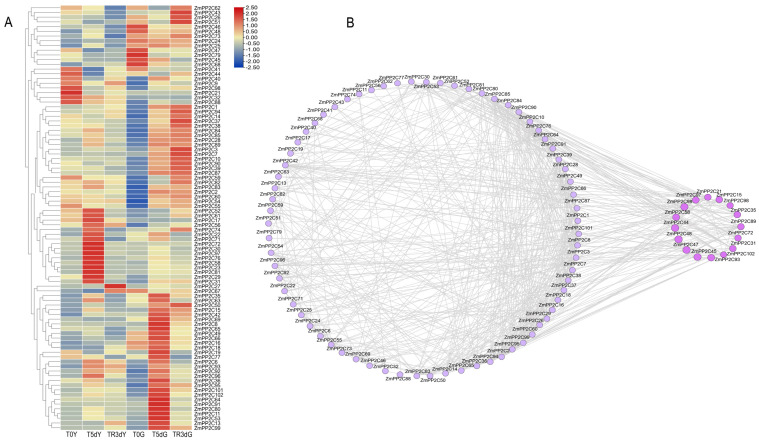
Gene co-expression network map and expression pattern analysis. (**A**) Analysis of *PP2C* gene expression in maize under drought-recovered water. In T0Y, T5dY, TR3dY, T0G, T5dG, and TR3dG, “Y” denotes leaf samples, “G” signifies root samples, and “T0, T5d, TR3d” represent pre-drought, 5 d after drought, and 3 d after rewatering, respectively. The red and green-blue gradients in the heatmap indicate an increase or decrease in gene FPKM values, respectively. (**B**) Co-expression network diagram analysis of maize *PP2C* genes. Lines in the graph indicate correlations between genes; circles in the graph represent *ZmPP2Cs* genes; and large purple circles indicate genes that are more highly correlated with other genes.

Cluster analysis of the expression of *ZmPP2C* family genes under drought-rewatering based on transcriptome data revealed that these genes showed different expression patterns under drought-rewatering conditions. Additionally, most of the *ZmPP2Cs* were involved in the response to drought-rewatering treatments (Figure 4A). The expression of *ZmPP2C15*/*23*/*29*/*30*/*81*/*72*/*97* increased significantly in the leaves after drought stress and decreased after rewatering, with the opposite pattern of expression in *ZmPP2C41/44/19/67.* The expression of *ZmPP2C6*/*40*/*45*/*77* was significantly increased in the roots under drought stress, subsequently decreasing after rewatering. In contrast, the *ZmPP2C82/59/62* genes displayed an opposite expression pattern in roots, decreasing under drought stress and increasing after rewatering. *ZmPP2C6* expression increased in both roots and leaves under drought stress and decreased after rewatering. In contrast, the expression of *ZmPP2C3*/*7*/*37* increased in the roots under drought stress and continued to increase after rewatering. These results suggest that these genes play important roles in drought stress and rewatering processes.

The correlation coefficients of 102 *ZmPP2Cs* were calculated, and genes with correlation coefficients greater than 0.75 were imported into Cytoscape to construct a gene co-expression network map (Figure 4B). *ZmPP2C15/21/29/31/35/44/45/47/48/58/72/89/93/97/98/102* had high K-values and belonged to the core node genes.

### 2.5. Expression Pattern Analysis of ZmPP2Cs in Response to Drought, ABA, and NaCl Treatments

**Figure 5:**
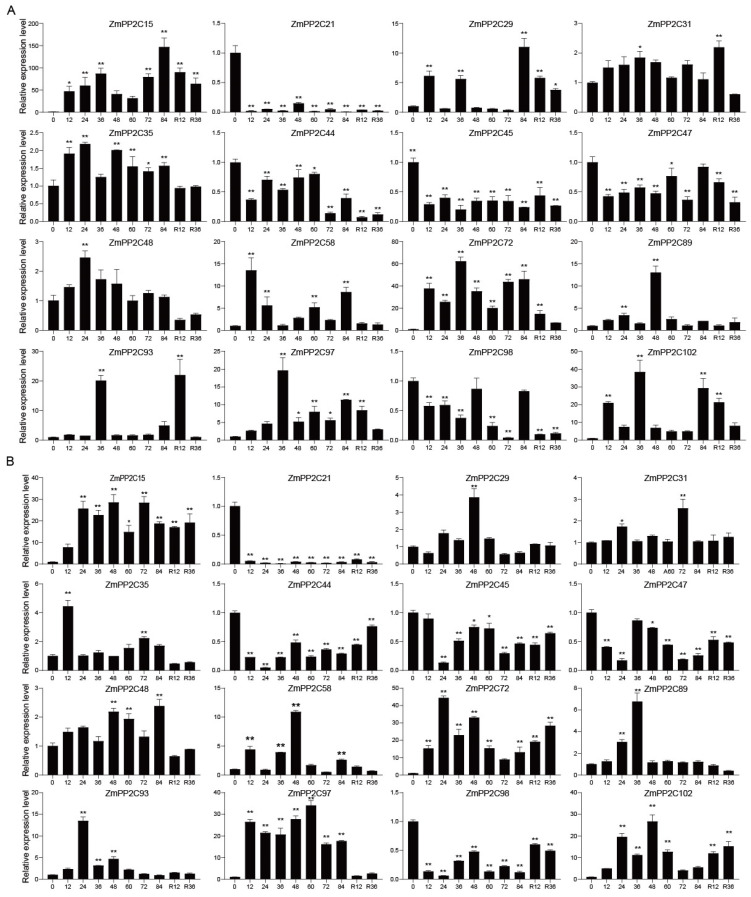
Expression levels of *ZmPP2C* genes under drought and ABA treatment. “h” denotes hours, and “R” signifies rewatering. (**A**) Drought, 20% PEG-6000 treatments; (**B**) ABA, 5 µ mol/L ABA treatment. Error bars show standard deviations (mean ± SD and *n* = 3), with asterisks indicating significant differences (ANOVA; * *p* < 0.05; ** *p* < 0.01).

Analysis of the expression patterns of *ZmPP2Cs* under drought, ABA, and NaCl treatments revealed that the expression of *ZmPP2C21*/*44*/*45*/*47*/*98* significantly decreased under drought stress (Figure 5A). *ZmPP2C15* expression peaked at 84 h and was 147 times higher than that before treatment. *ZmPP2C35*/*48*/*72*/*97*/*102* expression significantly increased under drought stress but decreased after rewatering. The expression of *ZmPP2C93* at 36 h was 20.15-fold higher than that before treatment, and the expression increased sharply after rewatering. Under ABA treatment (Figure 5B), *ZmPP2C29*/*31*/*35*/*48* expression increased slowly, and *ZmPP2C21*/*44*/*98* expression decreased significantly. The expression of *ZmPP2C15* peaked at 48 h, which was 28.55 times higher than that before treatment. The expression of *ZmPP2C72* peaked at 24 h, which was 46.33 times higher than before treatment. The expression of *ZmPP2C72* decreased with an increase in treatment time. Under NaCl treatment (Supplementary Figure S4), *ZmPP2C21*/*45*/*47* expression significantly decreased; in particular, *ZmPP2C21* expression after 48 h of stress was 0.04 times higher than that before stress. The expression of *ZmPP2C93* increased slowly and was 10.24-fold higher than that before treatment when normal growth conditions were restored for 36 h. *ZmPP2C15*/*97*/*102* genes were all significantly upregulated. The expression of *ZmPP2C15* after 84 h of treatment was 38.79 times higher than that before treatment. *ZmPP2C97* expression after 24 h of treatment was 23.18 times higher than that before treatment. After restoration to normal conditions, gene expression decreased but remained higher than before stress treatment.

In summary, *ZmPP2Cs* exhibited different expression patterns under drought, ABA, and NaCl stress. The expression of *ZmPP2C15/72/102* genes was significantly up-regulated under drought, ABA, and NaCl stress. Among them, *ZmPP2C15* showed the most significant amount of change under drought stress, indicating that *ZmPP2C15* plays an important role in drought stress.

### 2.6. Phenotypic Analysis of ZmPP2C15 Overexpression in A. thaliana under Drought Stress and Determination of Physiological Indexes

**Figure 6:**
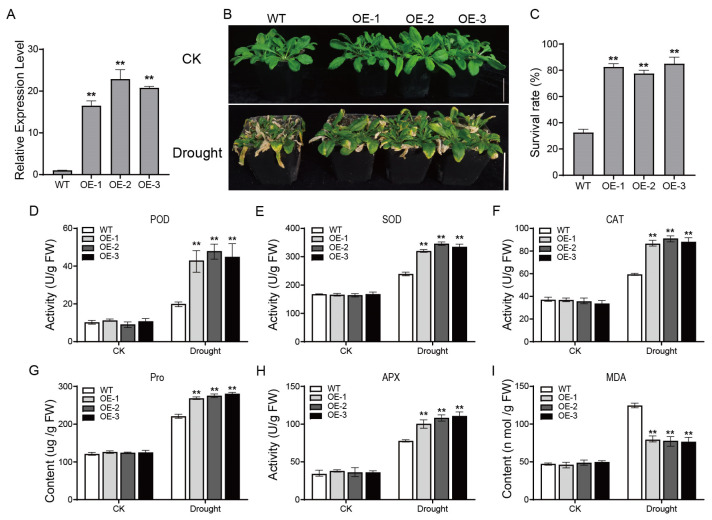
Phenotypic and physiological indexes of *Arabidopsis thaliana* overexpressing *ZmPP2C15* under drought stress. (**A**) *ZmPP2C15* expression in *Arabidopsis*. (**B**) Performance of WT and transgenic plants before and after natural drought treatment, Bar = 5 cm. (**C**) Survival of WT and transgenic plants under drought stress. (**D**) POD activity. (**E**) SOD activity. (**F**) CAT activity. (**G**) Pro content. (**H**) APX activity. (**I**) MDA content. Data represent mean ± SD of three replicates. ** indicates significant differences (*p* < 0.01) between WT and transgenic plants.

To investigate the function of *ZmPP2C15*, three transgenic *Arabidopsis* strains with *ZmPP2C15* overexpression (*OE-1*, *OE-2*, and *OE-3*) were selected (Figure 6A). Under normal conditions, the overexpression strain and wild-type (WT; Col) showed no obvious morphological differences in phenotype, consistent growth, or improved growth status. After drought stress, the growth of WT plants was severely inhibited, and the leaves dried, wilted, and died. However, overexpression plants were less affected and wilted to a lesser extent than the WT strains (Figure 6B). The survival rate of the overexpression plants was significantly higher than that of the WT strains (Figure 6C). This suggests that *ZmPP2C15* overexpression enhances drought tolerance in *A. thaliana*. Furthermore, relevant physiological and biochemical indexes were measured, and it was found that there was no significant difference between WT and overexpression plants under normal conditions in terms of MDA and Pro content, as well as SOD, POD, CAT, and APX activities (Figure 6D–I). After drought stress, Pro content and SOD, POD, CAT, and APX activities were higher in *ZmPP2C15* overexpression plants than in WT plants (*p* < 0.01), whereas MDA content was lower than that in WT plants (*p* < 0.01).

### 2.7. Analysis of Drought Tolerance in zmpp2c15 Maize Mutant Plants under Drought Stress

**Figure 7:**
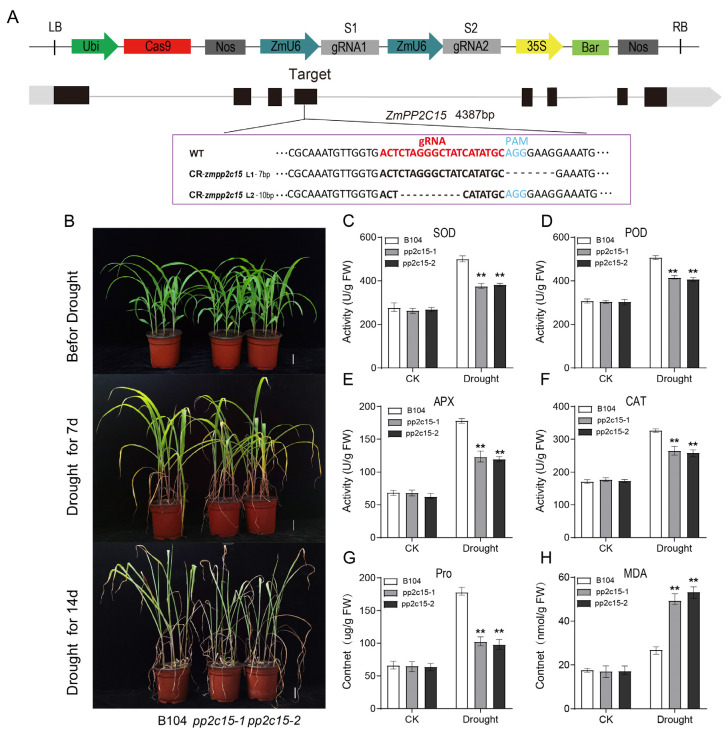
Phenotype and physiological indexes of the *ZmPP2C15* maize mutant under drought stress. (**A**) Knockout targets of *ZmPP2C15* mutant plants. (**B**) Phenotypic changes in B104 and mutant plants before and after natural drought treatment. Bar = 5 cm. (**C**) SOD activity. (**D**) POD activity. (**E**) APX activity. (**F**) CAT activity. (**G**) Pro content. (**H**) MDA content. Data represent mean ± SD of three replicates. ** indicates significant differences (*p* < 0.01) between WT and transgenic plants.

To further investigate the function of *ZmPP2C15*, two *zmpp2c15* mutant lines were generated by gene editing. Both the mutation sites were located in the fourth exon (Figure 7A). Under normal growth conditions, the *zmpp2c15* mutant and B104 plants grew in the same state but did not differ significantly. Under drought stress, both mutant and B104 plants exhibited wilting, curling of leaves, and yellowing of leaf tips. However, the wilting of mutant plants was more severe compared with that of B104 plants (Figure 7B). The growth status of both the mutant and B104 plants was severely affected by prolonged stress, but the mutant plants were more severely suppressed, indicating that *ZmPP2C15* knockdown reduced drought tolerance in maize. Physiological indexes were measured, and it was found that there were no significant differences in SOD, POD, APX, and CAT enzyme activities and Pro and MDA contents between the mutant and B104 strains before drought stress (Figure 7C–H). The SOD, POD, APX, and CAT enzyme activities and Pro content of the mutant plants were significantly lower than those of B104 after drought stress (*p* < 0.01), whereas the MDA content was significantly higher than that of B104. These results indicated that the *zmpp2c15* mutant had lower activities of osmoregulatory substances and antioxidant enzymes in vivo under drought stress, rendering maize more sensitive to drought.

### 2.8. Analysis of the Subcellular Localization and Protein Interactions of ZmPP2C15

**Figure 8:**
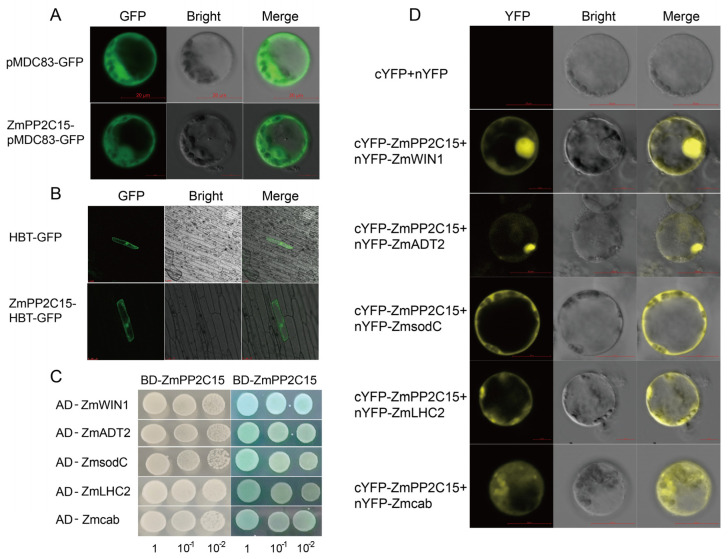
Analysis of subcellular localization and protein interactions of *ZmPP2C15*. (**A**) Fluorescent protein observed in maize protoplasts. (**B**) Fluorescent protein observed in onion epidermal cells. The fusion protein was instantaneously expressed in maize protoplasts under the control of the CaMV35S promoter and observed under a laser-scanning confocal microscope. Green represents GFP signal, and differential interference difference (DIC) images are shown. Scale bar = 20 μm. (**C**) Y2H, AD: pGADT7 vector; BD: pGBKT7 vector. 1, 10-1, 10-2: Yeast cells were coated at serial dilutions of 1:10, 1:100, and 1:1000 and cultured on SD/-Trp/-Leu and SD/-Ade/-His/-Leu/-Trp/X-a-gal media. (**D**) BiFC validation of reciprocal proteins of *ZmPP2C15*. cYFP: PXY-104 vector; nYFP: PXY-106 vector. Bar = 20 μm.

To further investigate the function and mechanism of action of *ZmPP2C15*, a *ZmPP2C15*-pMDC83-GFP fusion expression vector was constructed. This fusion vector was introduced into maize protoplasts, and observations were made using laser confocal microscopy. The results showed that the recombinant vector induced GFP expression in the cytoplasm and nucleus of the cells (Figure 8A). Similar green fluorescent proteins were observed in the cytoplasm and nucleus after a bombardment of onion epidermal cells using the gene gun method (Figure 8B). The results showed that the protein encoded by *ZmPP2C15* was localized to the cytoplasm and nucleus.

Using *ZmPP2C15* as a bait protein, we screened for reciprocal proteins in a Y2H system and identified nine proteins that interacted with *ZmPP2C15* (Supplementary Table S2). We co-transformed the reciprocal proteins with *ZmPP2C15* in yeast and found that proteins such as the ethylene-responsive transcription factor ZmWIN1, phenylalanine biosynthesis ZmADT2, Cu/Zn superoxide dismutase ZmsodC, light-trapping chlorophyll-binding protein Zmcab, and photosystem II light-trapping complex gene ZmLHC2 interacted with *ZmPP2C15*, as indicated by a blue color on the SD/-Ade/-His/-Leu/-Trp/X-a-gal plate (Figure 8C). Furthermore, we conducted a bimolecular fluorescence complementation (BiFC) assay and observed yellow fluorescent signals in maize protoplasts after the co-transformation of *ZmPP2C15* with ZmWIN1, ZmADT2, ZmsodC, Zmcab, and ZmLHC2 (Figure 8D). These results indicate that *ZmPP2C15* interacts with ZmWIN1, ZmADT2, ZmsodC, Zmcab, and ZmLHC2.

## 3. Discussion

Maize *PP2C* genes, evolving from the same ancestor, showed similarities in structure and function. A total of 102 maize *PP2C* genes were identified across the maize genome (Supplementary Table S1). Analysis of the positions of genes in this family on chromosomes revealed that 102 *ZmPP2Cs* were unevenly distributed on 10 chromosomes (Supplementary Figure S1). Studies on family gene evolution have revealed that tandem replication often leads to the formation of gene clusters [16]. Gene clusters in chromosome telomeres may participate in regulating chromosome replication and termination, thereby ensuring stable gene transmission and expression [17]. This study found that the majority of *ZmPP2C*s exist in gene clusters on different chromosomes or at different positions within the same chromosome, and *ZmPP2C*s mostly have gene clusters at the telomeres of chromosomes, indicating that members of the *ZmPP2C* family are conserved. Further analysis of the collinearity between maize, *Arabidopsis*, rice, and sorghum (Figure 3) revealed a replication relationship between maize and these species, emphasizing the strong conservatism of *ZmPP2C*s in the evolutionary process.

PP2C protein phosphatases are the largest family of protein phosphatases in plants and play important roles in plant growth and development, cell differentiation, and stress responses [18,19]. PP2C protein phosphatases of the A subfamily are capable of responding to drought stress and play important roles in signal transduction [10,20,21]. However, other PP2C subfamilies have been reported less frequently. In this study, we analyzed transcriptomic data and screened the *ZmPP2C15* gene of the F subfamily using gene co-expression network maps and expression patterns (Figure 4A,B). Using qRT-PCR and transcriptome data analysis, we found that *ZmPP2C15* was expressed in all maize tissues and organs (Attachments 2–3). Studies have previously established the expression of *OsPP2C09* and *TaPP2C-a10* in all tissues of rice and wheat [22,23], aligning with our findings. We found that the expression of *ZmPP2C15* was significantly upregulated under drought, ABA, and NaCl treatments, suggesting that *ZmPP2C15* is involved in the response to adversity stress (Figure 5A,B; Attachment 4). Moreover, *ZmPP2C55*, *ZmPP2C28*, and *ZmPP2C71* respond to drought, salt, high temperatures, and exogenous ABA treatments [18]. Furthermore, ramie *BnPP2C1*, *BnPP2C26*, and *BnPP2C27* respond to drought, high salt, and ABA treatments [24]. Thus, the results of the present study are consistent with those of the aforementioned studies.

In recent years, with the development of transgenic technology, many researchers have adopted transgenic plants to characterize gene functions. *TaPP2C-a10* acts as a negative regulator, as evidenced by *Arabidopsis* lines overexpressing *TaPP2C-a10*, which displayed early signs of wilting and death under drought stress [23]. Similarly, *CePP2C19* overexpression in *A. thaliana* resulted in less leaf yellowing and improved growth under drought stress, whereas silencing of *CePP2C19* significantly reduced drought tolerance in tiger nut plants [12]. In maize, *ZmPP2C55* overexpression lines exhibited better growth under drought stress, contrasting with the wilting and yellowing observed in WT plants [19]. In our study, we found that *Arabidopsis ZmPP2C15* overexpression plants were less affected by drought stress, and the plant growth status and survival rate were higher than those of plants overexpressing WT (Figure 6B,C). In contrast, *zmpp2c15* maize mutant plants were the first to wilt and exhibit dried leaves under drought stress (Figure 7B). The results indicate that *ZmPP2C15* can be induced to be expressed under drought stress, thereby playing a positive regulatory role. Our results align with those of previous studies, demonstrating that PP2C protein phosphatase can function under drought stress. As important protective enzymes in plant cells, antioxidant enzymes, whose activity increases significantly under drought stress, scavenge the accumulation of reactive oxygen species in plants, thereby maintaining cellular homeostasis [25,26]. SOD, POD, and CAT activities are higher in *ZmPP2C2* overexpressing tobacco plants under low-temperature stress [27]. Both POD and SOD activities are higher in *ZmPP2C55*-overexpressing maize plants than in WT plants under drought stress [19]. In this study, we found that POD, SOD, CAT, and APX activities were higher in *ZmPP2C15* overexpressing *Arabidopsi*s than in the WT strain under drought stress (Figure 6D–F). In contrast, *zmpp2c15* mutant plants had lower POD, SOD, CAT, and APX activities than those of the B104 plants (Figure 7C–F). These results indicated that *ZmPP2C15* overexpression enhanced the ability to scavenge reactive oxygen species in the plant, attenuated the degree of cell membrane damage, and enhanced the drought tolerance of the plant, which is consistent with the results of previous studies. Pro content, an osmoregulatory substance in the plant cytoplasm, is widely used to evaluate plant drought tolerance. In the present study, under drought stress, *ZmPP2C15* overexpressing Arabidopsis displayed a higher Pro content compared to WT, while *zmpp2c15* mutant plants exhibited a lower Pro content than B104 (Figure 6G and Figure 7G). MDA content responds to the degree of plant injury under drought stress. The MDA content in *ZmPP2C2* overexpressing tobacco plants was lower than that in WT plants under low-temperature stress [27]. Furthermore, MDA levels are lower in *ZmPP2C55*-overexpressing maize plants under drought stress [19]. In this study, we found that under drought stress, *ZmPP2C15* overexpressing *Arabidopsis* plants had a lower MDA content than that of WT plants (Figure 6I), whereas *zmpp2c15* mutant plants had a higher MDA content than those of B104 plants (Figure 7H). This indicated that *ZmPP2C15* overexpression increased drought tolerance in plants, which is consistent with the results of previous studies. These results indicate that *ZmPP2C15* responds to drought stress and acts as a positive regulator.

Additionally, subcellular localization results demonstrated that *ZmPP2C15* was situated in the nucleus and cytoplasm, consistent with previous studies on OsPP65 and AtPP2CG1 (Figure 8A,B). In addition, MdPP2C24/37, CePP2C19, GhPP2C1, and ZmPP2C85 are localized in the nucleus [27,28,29,30], and BpPP2C1 and GhPP1C2 are localized in the cell membrane and nucleus [31,32]. These results indicated that genes in the PP2C family exist in different locations from those in the cell, and their functions are different.

The Y2H assay is commonly used to screen target genes for reciprocal proteins, although it may produce false positives. BiFC enables intuitive and rapid detection of the localization of target proteins in cells and reciprocal proteins [33,34]. *PP2CAs* are the central components of ABA signaling and interact directly with ABA receptors and the protein kinase *SnRK2s* [19,35,36]. Interestingly, the F subfamily gene *ZmPP84* is not involved in the ABA signaling pathway but rather interacts with the MAPKK family member *ZmMEK1* [37]. In our study, we screened interacting proteins of *ZmPP2C15*, including the ethylene-responsive transcription factor ZmWIN1, phenylalanine biosynthesis factor ZmADT2, Cu/Zn superoxide dismutase ZmsodC, light-trapping chlorophyll-binding protein Zmcab, and photosystem II light-trapping complex ZmLHC2 (Figure 8C,D). We found that *ZmPP2C15* did not bind to PYL or SnRKs in response to drought stress, which is consistent with the results of a previous study [36]. This suggests that different *PP2C* family genes are involved in different regulatory pathways in response to drought stress. However, further investigation is needed regarding the specific response mechanism of *ZmPP2C15*.

In summary, the results of this study indicate that drought stress can significantly improve drought tolerance in transgenic *Arabidopsis* after *ZmPP2C15* overexpression. However, the drought tolerance of maize mutants decreased after the *ZmPP2C15* knockout. Therefore, we conclude that *ZmPP2C15* responds to drought stress and positively regulates under such conditions, offering potential for enhancing maize tolerance to drought stress and aiding in the breeding of new drought-resistant maize varieties. In the future, we will further reveal the detailed regulatory mechanisms of *ZmPP2C15*, such as protein phosphorylation and dephosphorylation, protein interactions, and potential protein modifications.

## 4. Material Methods

### 4.1. Identification of the Maize PP2C Gene Family

Maize protein sequences were downloaded from the Ensembl Plant database (http://plants.ensembl.org/Zea_mays/Info/Index, accessed on 11 April 2022). Hidden Markov model mapping of the PP2C protein structural domain (PF00481) was downloaded from the Pfam database (http://pfam.xfam.org/, accessed on 11 April 2022). The maize *ZmPP2C* gene was identified using HMMER3.0 (http://hmmer.org/download.html, accessed on 11 April 2022) with an E-value of <1 × 10^−5^. The resulting gene sequences were sequentially submitted to the SMART (http://smart.embl-heidelberg.de/, accessed on 12 April 2022), NCBI (https://www.ncbi.nlm.nih.gov/, accessed on 12 April 2022), and CDD (https://www.ncbi.nlm.nih.gov/cdd/term, accessed on 12 April 2022) websites for conserved structural domain analysis. Sequences that did not contain the complete structural domain of PP2C were eliminated to determine the final maize PP2C protein.

### 4.2. Bioinformatics Analysis of the ZmPP2C Gene Family

The obtained maize *PP2C* genes were sequentially named according to their positions on the chromosomes. The ExPASy ProtParam website (https://web.expasy.org/protparam/, accessed on 14 April 2022) was used to predict the amino acid number, isoelectric point, molecular mass, instability coefficient, hydrophilicity, and lipid coefficient of PP2C. WoLFPSORT (https://psort.hgc.jp/, accessed on 14 April 2022) was used to predict the subcellular localization of the PP2C proteins. Multiple sequence comparisons of *A. thaliana* and maize PP2C proteins were carried out using MEGA6.0 built-in Clustal Ww software, and a phylogenetic tree was constructed using the neighbor-joining (NJ) method [38]. The MEME website (http://meme-suite.org/tools/meme, accessed on 20 April 2022) was used to analyze the conserved motifs of the ZmPP2C proteins, with a maximum motif output value of 20 [39]. TBtools was used to visualize the gene structure of *ZmPP2Cs*. The locations of the maize *PP2C* gene family members on the chromosomes were mapped using the online mapping tool MG2C (http://mg2c.iask.in/mg2c_v2.0/, accessed on 21 April 2022). The TBtools MCscan algorithm was used to analyze the synteny of *PP2C* genes in maize, *Arabidopsis*, sorghum, and rice.

### 4.3. Expression Profiling and Co-Expression Network Map Analysis of ZmPP2Cs

A local BLAST search of 102 *ZmPP2Cs* was performed using transcriptome data of maize seedling leaves (T0Y, T5dY, and TR3dY) and roots (T0G, T5dG, and TR3dG) under drought stress and rewatering treatments. Differentially expressed genes of *ZmPP2Cs* were screened based on stringent criteria (│Log2 (fold change)│ ≥ 2, FDR < 0.05 as screening conditions). We used FPKM values based on transcriptome data to map gene expression patterns into heat maps using the TBtools. To screen for core node genes, we imported correlation coefficients greater than 0.75 into Cytoscape 3.8.2 software to map gene co-expression networks [40].

### 4.4. Plant Growing Conditions and Treatments

Maize B104, characterized by full and uniformly sized grains, was chosen for testing. Planted in nutrient soil, it was cultivated in a light incubator (28 °C, 16 h light/8 h dark). When the corn grew to the three-leaf stage, we applied a drought-rewatering treatment to the corn seedlings with the same growth. We measured the soil moisture content using a soil temperature and humidity meter (SYS-WSD, Changchun, China), which ranged from 45% to 50% during drought stress and 98% when watering was resumed. Root and leaf samples were collected before drought stress, at 5 d of stress (T0Y, T5dY), and at 3 d of rewatering (TR3dY); and similarly, for grain samples (T0G, T5dG, TR3dG). Transcriptome sequencing was performed with five plants as samples, each with three biological replicates.

Maize B73 was used as the study material and cultured in a light incubator (28 °C, 16 h light/8 h dark). Uniformly growing maize seedlings at the three-leaf stage were subjected to stress: 20% PEG-6000 for drought stress, 200 mmol/L NaCl for salt stress, and 5 μmol/L ABA for ABA treatment. Leaf tissues were randomly selected at various time points (0, 12, 24, 36, 48, 60, 72, and 84 h) and at 12 and 36 h after stress relief and stored at −80 °C. Three biological replicates were used for each treatment.

### 4.5. RNA Extraction, cDNA Synthesis, and qRT-PCR Analysis

Corn leaves (0.1 g) were ground in liquid nitrogen, and the total RNA was extracted using a TRIzol extraction kit (Invitrogen, Beijing, China). We used a concentration meter to detect the concentration and purity of RNA and 1% agarose gel electrophoresis to detect total RNA quality. Subsequently, 1 μg of total RNA was taken and reverse transcribed into cDNA (Prime-ScriptTM RT reagent kit with gDNA Eraser, TaKaRa, Beijing, China). The resulting cDNA was diluted to a final concentration of 100 μg·μL^−1^, serving as a template for gene amplification in subsequent qRT-PCR analyses. Primers were designed using NCBI for the 16 screened *ZmPP2Cs*, with maize actin 18S as an internal reference. The primers are shown in Supplementary Table S3. The diluted cDNA was used as a template and analyzed via qRT-PCR using the Hieff qPCR SYBR Green Master Mix (No Rox) (YEASEN, Shanghai, China). The reaction mixture consisted of 10 μL of enzyme (Hieff qPCR SYBR Green Master Mix), 0.4 μL of each primer, 1 μL of cDNA, and 8.2 μL of ddH_2_O. The reaction program comprised pre-denaturation at 95 °C for 5 min, denaturation at 95 °C for 10 s, and annealing at 60 °C for 30 s for 40 cycles. The relative gene expression was calculated using the 2^−∆∆CT^ method, with three replicates per treatment [41]. The relative expression level (2^−∆∆Ct0 h^) of untreated control plants was categorized as 1. 

### 4.6. Construction of Arabidopsis Overexpression Vectors, Obtaining Positive Plants, and Verifying Drought Tolerance

We constructed the overexpression vector *ZmPP2C15-pFGC5941* and used the *Agrobacterium* infiltration inflorescence method to infiltrate *A. thaliana*, obtaining three independent transgenic lines. Seeds overexpressing pure *ZmPP2C15* and WT seeds were sterilized with 75% alcohol and coated in 1/2 MS medium for one week. Seedlings with uniform growth were transferred to nutrient soil for incubation and subjected to natural drought stress (soil water content 45–50%) when they grew five leaves. Phenotypic changes and measured physiological indexes were observed.

### 4.7. Construction of Maize Mutant Vectors, Obtaining Mutant Plants, and Verifying Drought Tolerance

The shear target of *ZmPP2C15* was selected (website: http://www.genome.arizona.edu/crispr/CRISPRsearch.html, accessed on 5 September 2022), and the primers used are listed in Supplementary Table S3. Next, we amplified the sgRNA, purified the recovered PCR product, and constructed the CRISPR/Cas9 vector pBUE411 for *ZmPP2C15*. The correctly sequenced bacteriophage was extracted, and the plasmid was transferred into *Agrobacterium* GV3101 for the genetic transformation of maize. After obtaining the mutant seeds for target site detection, B104 and pure mutant seeds were sown in a greenhouse. When the soil moisture content reached 80%, natural drought stress treatment was applied until the soil moisture content reached 45%. Subsequently, changes in the phenotypes of the B104 and *zmpp2c15* mutants were analyzed for physiological and biochemical indexes.

### 4.8. Subcellular Localization of ZmPP2C15

A fusion expression vector of *ZmPP2C15*-pMDC83 green fluorescent protein (GFP) was constructed. Protoplasts were extracted using yellowing seedlings of maize B73, and the recombinant plasmids were introduced into the protoplasts using the PEG4000 method and then cultured in the dark at 28 °C for 12–18 h [42]. The subcellular localization was observed using a laser confocal microscope (Zeiss LSM980, Oberkochen, Germany). A transient expression vector, *ZmPP2C15*-HBT, was constructed. We used the gene gun method to bombard onion epidermal cells [43]. The bombarded onion epidermis was cultured in hypertonic medium for about 12 h and then transferred to MS medium and cultured at 28 °C for 12 h. Subcellular localization was observed using a laser confocal microscope (Zeiss LSM980).

### 4.9. Validation of Protein Interactions Using Yeast Two-Hybrid (Y2H) and BiFC

For Y2H experiments, we constructed *ZmPP2C15*- pGBKT7 fusion in expression vectors. Toxicity and self-activation assays were performed using *ZmPP2C15*. Transformation was performed using the Matchmaker Gold Y2H System, and proteins that interacted with *ZmPP2C15* were screened.

The expression vector *ZmPP2C15*-PXY104 was constructed, while *ZmWIN1*, *ZmADT2*, *ZmsodC*, *Zmcab*, and *ZmLHC2* were constructed using vector PXY106. The fusion expression vector was introduced into maize protoplasts and cultured in the dark at 28 °C for 12–18 h. Finally, the fluorescence signal was observed under a laser confocal microscope (Zeiss LSM980).

### 4.10. Measurement of Physiological Indicators

Free proline (Pro) content was determined using the acid ninhydrin method. The SOD activity was determined using the nitro-blue tetrazolium method. The POD activity was determined using the Guaiacol method. The MDA content was determined using the thiobarbituric acid method. Finally, the CAT content was determined using the potassium permanganate titration method, and the APX content was determined using ultraviolet spectrophotometry [44].

### 4.11. Statistical Analysis

All experiments were performed in triplicate. We used Excel 2010 for data processing, Graphpad Pism 9.5 for graphing, and SPSS 22 to analyze the data for significant differences. The data were presented as the mean ± standard deviation (SD). Statistical analysis employed the Student’s *t*-test, with * *p* ≤ 0.05 and ** *p* ≤ 0.01 denoting significance.

## 5. Conclusions

In the present study, 102 *ZmPP2Cs* were identified in the maize genome, and comprehensive analyses of their structural features, phylogeny, and expression profiles were conducted. Sixteen core genes responding to drought stress were screened, and their expression changes under drought and ABA treatments were analyzed. In addition, the knockout of *ZmPP2C15* reduced the enzyme activity of SOD, POD, APX, and CAT, as well as Pro content, while increasing MDA content, resulting in weakened drought tolerance in maize. However, after overexpression of *ZmPP2C15*, the trend of changes in these indicators was the opposite and improved the drought tolerance of *Arabidopsis*. The subcellular localization of *ZmPP2C15* in the nucleus and cytoplasm, along with its interaction with various proteins, including ZmWIN1 and ZmADT2, provides valuable insights into the evolution and functional aspects of the *ZmPP2C* gene family in maize and holds significance for the development of new drought-resistant maize varieties.

## Data Availability

The datasets presented in this paper can be found in online repositories. The names of the repository/repositories and accession number(s) can be found in BioProject. The BioProject ID is PRJNA942991.

## Supplementary Materials

The following supporting information can be downloaded at https://www.mdpi.com/article/10.3390/plants13030340/s1, Supplementary Figure S1: Location of maize PP2C gene family members on the chromosome; Supplementary Figure S2: Expression patterns of *ZmPP2Cs* genes in various organs and under different stresses in maize; Supplementary Figure S3: Expression of *ZmPP2Cs* genes in different tissues of maize; Supplementary Figure S4: Expression of *ZmPP2Cs* gene under NaCl treatment; Supplementary Table S1: Maize PP2C gene family member information; Supplementary Table S2: Functional analysis of pGBKT7-*ZmPP2C15* interacting proteins; Supplementary Table S3: Information on all primers covered in the article.
