# Supplementary material for: Identification of the Maize PP2C Gene Family and Functional Studies on the Role of ZmPP2C15 in Drought Tolerance
Source: Plants (Basel). 2024 Jan 23;13(3):340. doi: 10.3390/plants13030340 (PMC10856965; doi:10.3390/plants13030340)
Supplement: Supplementary file 1 [file plants-13-00340-s001.zip › Supplementary Table S1.pdf]

Supplementary Table S1: Maize PP2C gene family member information

Gene ID	Gene name	Chr	Amino acid	Isoelectric point	Molecular weight(kD)	Instability Index	GRAVY	Aliphatic index	Subcellular localization
Zm00001eb003000	ZmPP2C1	1	357	7.25	39.94	45.90	- 0.323	85.71	chlo
Zm00001eb003010	ZmPP2C2	1	399	8.49	44.15	48.18	**- 0.316**	83.31	chlo
Zm00001eb003020	ZmPP2C3	1	399	8.69	44.06	48.16	- 0.309	82.83	chlo
Zm00001eb012040	ZmPP2C4	1	296	5.40	31.14	54.40	- 0.241	77.80	nucl
Zm00001eb012350	ZmPP2C5	1	623	6.09	67.78	45.57	- 0.350	72.87	nucl
Zm00001eb013450	ZmPP2C6	1	388	5.97	40.79	60.55	- 0.196	80.08	chlo
Zm00001eb018090	ZmPP2C7	1	847	5.82	93.10	54.81	- 0.573	69.00	nucl
Zm00001eb019240	ZmPP2C8	1	440	5.60	47.35	40.37	-0.222	85.32	nucl
Zm00001eb022330	ZmPP2C9	1	375	6.07	40.36	55.21	- 0.201	79.57	nucl
Zm00001eb034100	ZmPP2C10	1	515	5.27	55.04	42.56	- 0.372	76.17	nucl
Zm00001eb058130	ZmPP2C11	1	376	9.39	41.02	40.05	- 0.256	87.26	chlo
Zm00001eb058920	ZmPP2C12	1	377	9.15	43.09	41.19	- 0.548	74.51	mito
Zm00001eb062680	ZmPP2C13	1	615	5.71	66.28	49.22	-0.259	79.84	chlo
Zm00001eb063490	ZmPP2C14	1	233	5.29	24.23	38.75	0.019	92.62	chlo
Zm00001eb067610	ZmPP2C15	2	284	4.70	30.66	33.89	-0.227	80.28	cyto
Zm00001eb071240	ZmPP2C16	2	315	8.72	34.15	40.17	-0.364	77.08	chlo
Zm00001eb072840	ZmPP2C17	2	392	6.18	43.68	44.13	-0.253	90.08	cyto
Zm00001eb081430	ZmPP2C18	2	444	5.80	47.36	50.22	-0.220	80.70	mito
Zm00001eb084510	ZmPP2C19	2	521	4.86	57.47	44.28	-0.202	85.68	chlo
Zm00001eb088070	ZmPP2C20	2	355	8.05	38.42	76.65	-0.400	65.77	cyto
Zm00001eb094040	ZmPP2C21	2	473	5.17	51.02	41.99	-0.434	75.29	cyto
Zm00001eb098220	ZmPP2C22	2	350	6.34	36.96	62.00	-0.216	76.60	nucl
Zm00001eb105950	ZmPP2C23	2	306	6.03	33.34	42.08	-0.536	79.08	cyto
Zm00001eb107130	ZmPP2C24	2	298	9.45	32.12	48.63	-0.290	73.39	chlo
Zm00001eb110450	ZmPP2C25	2	454	5.03	47.48	47.05	- 0.099	79.01	chlo
Zm00001eb111860	ZmPP2C26	2	364	6.47	38.92	41.94	- 0.100	93.79	chlo
Zm00001eb112250	ZmPP2C27	2	429	6.96	44.57	45.08	0.130	95.38	chlo
Zm00001eb132080	ZmPP2C28	3	484	7.55	51.51	42.94	-0.171	87.11	chlo
Zm00001eb148130	ZmPP2C29	3	408	5.74	43.28	58.76	-0.202	77.77	nucl
Zm00001eb158020	ZmPP2C30	3	410	5.81	43.43	48.54	-0.230	80.85	nucl
Zm00001eb160470	ZmPP2C31	3	472	4.60	49.29	46.41	0.161	90.95	vacu
Zm00001eb162530	ZmPP2C32	3	384	5.43	41.18	39.44	-0.006	95.05	chlo
Zm00001eb168010	ZmPP2C33	4	436	6.27	45.45	50.66	0.094	92.06	chlo
Zm00001eb181140	ZmPP2C34	4	458	6.24	49.96	38.13	-0.379	79.19	nucl
Zm00001eb182690	ZmPP2C35	4	449	6.07	48.21	58.85	-0.268	80.13	vacu
Zm00001eb192230	ZmPP2C36	4	272	4.92	29.48	35.77	-0.318	73.49	cyto
Zm00001eb199570	ZmPP2C37	4	523	5.12	56.37	41.37	-0.386	75.39	chlo
Zm00001eb203040	ZmPP2C38	4	465	7.64	49.27	38.12	-0.153	82.71	chlo
Zm00001eb204210	ZmPP2C39	4	240	4.75	26.28	38.81	-0.267	86.58	cyto
Zm00001eb205090	ZmPP2C40	4	382	5.24	40.62	46.59	-0.314	73.61	chlo

Zm00001eb208010	ZmPP2C41	4	290	4.76	31.21	26.65	-0.400	78.38	cysk
Zm00001eb211690	ZmPP2C42	5	632	5.41	68.20	51.50	-0.279	76.96	chlo
Zm00001eb228190	ZmPP2C43	5	394	8.94	43.80	49.15	-0.218	89.52	chlo
Zm00001eb232360	ZmPP2C44	5	348	4.90	37.39	31.45	-0.243	84.66	cyto
Zm00001eb234050	ZmPP2C45	5	359	5.22	39.22	33.1	-0.371	74.74	nucl
Zm00001eb238250	ZmPP2C46	5	298	5.00	32.48	33.63	-0.210	86.14	cyto
Zm00001eb239920	ZmPP2C47	5	385	6.35	41.16	44.68	-0.391	73.82	nucl
Zm00001eb240940	ZmPP2C48	5	418	8.82	44.72	43.68	-0.197	79.90	mito
Zm00001eb244550	ZmPP2C49	5	446	5.79	47.99	58.66	-0.285	79.75	vacu
Zm00001eb250950	ZmPP2C50	5	345	9.63	38.52	43.95	-0.226	93.62	chlo
Zm00001eb251160	ZmPP2C51	5	570	5.57	62.08	51.69	-0.273	81.88	nucl
Zm00001eb258010	ZmPP2C52	5	354	5.23	39.21	41.31	-0.412	75.73	cyto
Zm00001eb264360	ZmPP2C53	6	431	7.12	45.09	44.19	0.015	86.33	chlo
Zm00001eb272240	ZmPP2C54	6	390	9.03	43.14	50.55	-0.243	88.72	chlo
Zm00001eb274970	ZmPP2C55	6	365	5.00	40.08	35.36	-0.404	73.26	chlo
Zm00001eb285830	ZmPP2C56	6	419	9.13	45.52	49.46	-0.333	77.52	chlo
Zm00001eb288530	ZmPP2C57	6	679	9.88	75.58	53.11	-0.246	85.46	plas
Zm00001eb294200	ZmPP2C58	6	284	5.23	30.18	38.25	0.067	89.01	chlo
Zm00001eb297300	ZmPP2C59	6	356	4.74	37.40	42.84	-0.050	81.46	chlo
Zm00001eb297570	ZmPP2C60	6	429	4.58	46.94	49.69	-0.601	69.16	cysk
Zm00001eb303240	ZmPP2C61	7	583	7.03	63.73	51.52	-0.353	82.13	chlo
Zm00001eb309620	ZmPP2C62	7	338	6.28	36.47	37.31	-0.419	77.07	nucl
Zm00001eb309920	ZmPP2C63	7	358	6.38	37.60	53.91	-0.142	78.10	nucl
Zm00001eb315010	ZmPP2C64	7	431	6.06	47.16	46.58	-0.190	84.11	chlo
Zm00001eb320490	ZmPP2C65	7	363	4.96	39.54	37.21	-0.452	75.21	chlo
Zm00001eb322340	ZmPP2C66	7	290	6.27	31.73	28.63	-0.371	87.38	cyto
Zm00001eb324540	ZmPP2C67	7	431	5.72	46.36	44.42	-0.286	75.41	chlo
Zm00001eb329360	ZmPP2C68	7	352	5.00	37.24	38.57	-0.056	82.76	cyto
Zm00001eb331410	ZmPP2C69	7	429	7.51	46.02	41.95	-0.193	86.18	chlo
Zm00001eb332150	ZmPP2C70	8	396	5.01	43.05	58.93	-0.119	90.38	cyto
Zm00001eb342010	ZmPP2C71	8	255	5.91	28.08	35.43	-0.139	89.88	cysk
Zm00001eb344210	ZmPP2C72	8	296	7.72	31.73	69.21	-0.353	71.89	nucl
Zm00001eb345020	ZmPP2C73	8	429	4.54	46.75	48.17	-0.581	71.82	nucl
Zm00001eb345270	ZmPP2C74	8	423	5.02	44.90	44.71	-0.091	84.73	chlo
Zm00001eb355760	ZmPP2C75	8	508	5.92	54.39	47.15	-0.300	79.00	chlo
Zm00001eb356420	ZmPP2C76	8	391	5.57	41.76	37.54	-0.147	89.36	chlo
Zm00001eb357000	ZmPP2C77	8	473	8.90	50.38	39.87	-0.237	78.84	chlo
Zm00001eb357040	ZmPP2C78	8	440	4.91	46.57	39.56	-0.008	84.70	chlo
Zm00001eb358340	ZmPP2C79	8	258	5.33	28.11	45.27	-0.131	85.81	cyto
Zm00001eb359460	ZmPP2C80	8	300	8.83	31.02	62.31	-0.207	78.10	nucl
Zm00001eb367840	ZmPP2C81	8	413	6.34	43.82	52.84	-0.218	75.91	nucl
Zm00001eb374920	ZmPP2C82	9	354	5.05	39.03	35.49	-0.256	80.40	cyto
Zm00001eb385910	ZmPP2C83	9	360	5.09	39.09	52.49	-0.206	75.67	cyto

Zm00001eb388780	ZmPP2C84	9	365	4.90	40.21	34.61	-0.417	75.67	cyto
Zm00001eb388790	ZmPP2C85	9	366	4.70	40.15	37.93	-0.419	75.74	cyto
Zm00001eb392360	ZmPP2C86	9	475	6.47	52.68	44.02	-0.045	95.92	plas
Zm00001eb392700	ZmPP2C87	9	437	5.24	46.92	37.12	-0.211	86.34	nucl
Zm00001eb393810	ZmPP2C88	9	372	6.40	40.29	54.49	-0.144	83.36	cyto
Zm00001eb393920	ZmPP2C89	9	393	8.51	43.59	45.00	-0.339	82.06	chlo
Zm00001eb394120	ZmPP2C90	9	964	5.68	10.61	47.61	-0.557	69.32	nucl
Zm00001eb396460	ZmPP2C91	9	430	6.53	45.91	38.07	-0.216	80.93	nucl
Zm00001eb396970	ZmPP2C92	9	388	5.82	40.94	60.19	-0.181	81.80	chlo
Zm00001eb400570	ZmPP2C93	9	391	8.03	42.14	49.92	-0.178	86.55	cyto
Zm00001eb402720	ZmPP2C94	9	399	8.69	44.15	44.13	-0.332	83.08	chlo
Zm00001eb413940	ZmPP2C95	10	325	6.46	35.11	54.72	-0.461	72.34	chlo
Zm00001eb417220	ZmPP2C96	10	405	4.82	44.30	59.30	-0.176	82.81	chlo
Zm00001eb419870	ZmPP2C97	10	357	6.04	37.92	67.74	-0.340	67.09	nucl
Zm00001eb421700	ZmPP2C98	10	521	4.90	57.18	43.55	-0.190	85.49	chlo
Zm00001eb423600	ZmPP2C99	10	428	5.68	45.82	50.27	-0.297	79.16	chlo
Zm00001eb425880	ZmPP2C100	10	269	5.60	29.16	38.13	-0.219	81.71	cyto
Zm00001eb430620	ZmPP2C101	10	318	8.73	34.55	40.93	-0.378	76.70	chlo
Zm00001eb431460	ZmPP2C102	10	284	5.83	31.19	29.15	-0.254	87.54	cyto

Note: Nucl. Nucleus; Cyto. Cytoplasm; Chlo. Chloroplast; Pero. Peroxisome; Mito. Mitochondrion; Extr. Extracellular; Plas. Plasma membrane
