# Supplementary material for: Identification of the Maize PP2C Gene Family and Functional Studies on the Role of ZmPP2C15 in Drought Tolerance
Source: Plants (Basel). 2024 Jan 23;13(3):340. doi: 10.3390/plants13030340 (PMC10856965; doi:10.3390/plants13030340)
Supplement: Supplementary file 1 [file plants-13-00340-s001.zip › Supplementary Table S2.pdf]

Supplementary Table S2: Functional analysis of pGBKT7-ZmPP2C15 interacting proteins

Prey number	Gene ID	Gene Function Annotation
1	Zm00001d046501	Ethylene-responsive transcription factor WIN1
2	GRMZM2G115841	Phenylalanine biosynthesis II
3	GRMZM2G148074	Homology box leucine zipper protein HD-ZIP protein
4	GRMZM2G106928	Copper/zinc superoxide dismutase
5	GRMZM2G413897	16.9 kDa class I heat shock protein 2
6	GRMZM2G402936	Photosystem II light-trapping complex gene
7	GRMZM2G057281	Phototrap chlorophyll B binding protein
8	GRMZM2G057571	Zinc Knuckle (CCHC-type) Family Proteins
9	GRMZM2G153068	Chaperone protein dnaJ 1, mitochondria-like
