# Supplementary material for: Identification of the Maize PP2C Gene Family and Functional Studies on the Role of ZmPP2C15 in Drought Tolerance
Source: Plants (Basel). 2024 Jan 23;13(3):340. doi: 10.3390/plants13030340 (PMC10856965; doi:10.3390/plants13030340)
Supplement: Supplementary file 1 [file plants-13-00340-s001.zip › Supplementary Table S3.pdf]

Supplementary Table S3: Information on all primers covered in the article

Primer	Sequences（F:5' to 3';R: 3' to 5'）
DL005071-F	TCTGATCCCTGTCCAGCTCA
DL005071-R	TGAGGCAGAAGTCACCGAAC
DL042886-F	CCGCGTCATTTACTGGGATG
DL042886-R	GATCAGACACTCGTCGTCGT
DL044301-F	CCATGACGTTCCGCTTACCT
DL044301-R	ATCGTGTTTCCCCCAACCTC
DL000098-F	GCCAAAATGATATGGCTTGTCT
DL000098-R	GATGTAAGCACCTAGGCCTAAT
DL015279-F	AAGAATTGAGGACGCAGGGG
DL015279-R	ATCACCAAATGCGCGAGAGA
DL015504-F	TATCCACGAGCGTATAAACACG
DL015504-R	TTTTGAACTGCACCAGGATCAT
DL016332-F	AAGCAGGGGAAGGACCTCAT
DL016332-R	CGCTTGATACGCTCAGCCTC
DL016474-F	CATTCCGTGTAATGGATAGGGA
DL016474-R	TTCGCTTGGAATGTTAGGTTTG
DL038846-F	CTGCTCTGCCTGCTTCTTTC
DL038846-R	GGTATGGCTACAGAGTACAGTC
DL009626-F	CTGAGACTGAGTTGAGTTGAGT
DL009626-R	CACACGCTGCTCAAATACTTAA
DL047385-F	GTCCAAAGTAGCCCGCGTAA
DL047385-R	CAGCAGTTGTCATGGCGTTT
DL048131-F	GGTGACTACTCCATCGCCGT
DL048131-R	GCCTTCTGGAACACCTCCG
DL025055-F	ATCGCTGAGCCGGAGATCA
DL025055-R	GATGTCGTTGCTGATCACGTC
DL025323-F	CTCGTGCTTTTGGAGTTGGC
DL025323-R	ATGGAGGGCTGCTCAAATCG
DL026363-F	GATCTGGGACAAGGTGGCTC
DL026363-R	ACTGACCACATACTCGGGGA
18s-F	CCTGCGGCTTAATTTGACTC
18s-R	GTTAGCAGGCTGAGGTCTCG

5941-PP2C15-F	AGGCGCGCCAATGGGGTACTTGAGCTCCGT
5941-PP2C15-R	CGGGATCCTTAAGCGTATCCGCTGCTAC
Cas9-PP2C15F	AATAATGGTCTCAGGCGACTCTAGGGCTATCATATGC
Cas9- PP2C15F0	GACTCTAGGGCTATCATATGCGTTTTAGAGCTAGAAATAGC
Cas9-PP2C15R0	GCATATGATAGCCCTAGAGTCGCTTCTTGGTGCC
Cas9- PP2C15R	ATTATTGGTCTCTAAACGCATATGATAGCCCTAGAGT
HBT- PP2C15-F	GGGGTACCATGGGGTACTTGAGCTCCGT
HBT- PP2C15-R	TCCCCCCGGGGGAGCGTATCCGCTGCTACCTTGCC
pMDC83- PP2C15-F	GGACTAGTATGGGGTACTTGAGCTCCGT
pMDC83- PP2C15- R	CGGGATCCAGCGTATCCGCTGCTACCTT
BD-PP2C15-F	TGCATATGGCCATGGAGGCCGAATTCATGGGGTACTTGAGCTCCGT
BD-PP2C15-R	TGCGGCCGCTGCAGGTCGACGGATCCTTAAGCGTATCCGCTGCTAC
PGADT7-1	CTATTCGATGATGAAGATACCCCACCAAACCCA
PGADT7-2	GTGAACTTGCGGGGTTTTTCAGTATCTACGATT
AD-046501-F	ATGGAGGCCAGTGAATTCATGGTGCAATCAAAGAAGAAG
AD-046501-R	CTCGAGCTCGATGGATCCTCAGATGACGAGGCTGCCTG
AD-115841-F	ATGGAGGCCAGTGAATTCATGGCCTACACCTCCTCCCT
AD-115841-R	CTCGAGCTCGATGGATCCTCACTGCAGATCGTAGACGG
AD-148074-F	ATGGAGGCCAGTGAATTCATGGATATTATGGCGCTTAATGCG
AD-148074-R	CTCGAGCTCGATGGATCCTCACGGCCTCCGGGCTGGGCCGT
AD-106928-F	ATGGAGGCCAGTGAATTCATGGCCGCGCAGTCCTTCCT
AD-106928-R	CTCGAGCTCGATGGATCCCTACAATGGAGTCAGGCCAAC
AD-413897-F	ATGGAGGCCAGTGAATTCATGTCTCTGGTAAGGCTCCTG
AD-413897-R	CTCGAGCTCGATGGATCCGTCGAGGCCGGCCCCTGCTGA
AD-402936-F	ATGGAGGCCAGTGAATTCATGGCGAGCAGCACCATGGCC
AD-402936-R	CTCGAGCTCGATGGATCCTCAGCGAGGTTCTCGAGCGGGC
AD-057281-F	ATGGAGGCCAGTGAATTCATGGCGTCGACGATCATGGC
AD-057281-R	CTCGAGCTCGATGGATCCTTAAGACCCTGGTGCGAACT
AD-057571-F	ATGGAGGCCAGTGAATTCATGGCCGCGCCGCCGCCCACGGGCT
AD-057571-R	CTCGAGCTCGATGGATCCCTAGGTGGGCTGCTGCGAGGCA
AD-153068-F	ATGGAGGCCAGTGAATTCATGTCTGGCAGTAGCCCTGCA
AD-153068-R	CTCGAGCTCGATGGATCCCTATCCCCCCGATGCTCCAGCT
104- PP2C15-F	ATTACAGGTACCCGGGGATCCATGGGGTACTTGAGCTCCGT
104- PP2C15-R	CACGCTGCCACCGCCGTCGACAGCGTATCCGCTGCTACCTTGCCC
106-046501-F	ATCGAGGACGCCGGCGGATCCATGGTGCAATCAAAGAAGAAG
106-046501-R	GAACGAAAGCTCTGCAGGTCGACTCAGATGACGAGGCTGCCTG
106-115841-F	ATCGAGGACGCCGGCGGATCCATGGCCTACACCTCCTCCCT
106-115841-R	GAACGAAAGCTCTGCAGGTCGACTCACTGCAGATCGTAGACGG

106-106928-F	ATCGAGGACGCCGGCGGATCCATGGCCGCGCAGTCCTTCCT
106-106928-R	GAACGAAAGCTCTGCAGGTCGACCTACAATGGAGTCAGGCCAAC
106-402936-F	ATCGAGGACGCCGGCGGATCCATGGCGAGCAGCACCATGGCC
106-402936-R	GAACGAAAGCTCTGCAGGTCGACTCAGCGAGGTTCTCGAGCGGGC
106-057281-F	ATCGAGGACGCCGGCGGATCCATGGCGTCGACGATCATGGC
106-057281-R	GAACGAAAGCTCTGCAGGTCGACTTAAGACCCTGGTGCGAACT
