# Supplementary figures and images for: Identification of the Maize PP2C Gene Family and Functional Studies on the Role of ZmPP2C15 in Drought Tolerance
Source: Plants (Basel). 2024 Jan 23;13(3):340. doi: 10.3390/plants13030340 (PMC10856965; doi:10.3390/plants13030340)

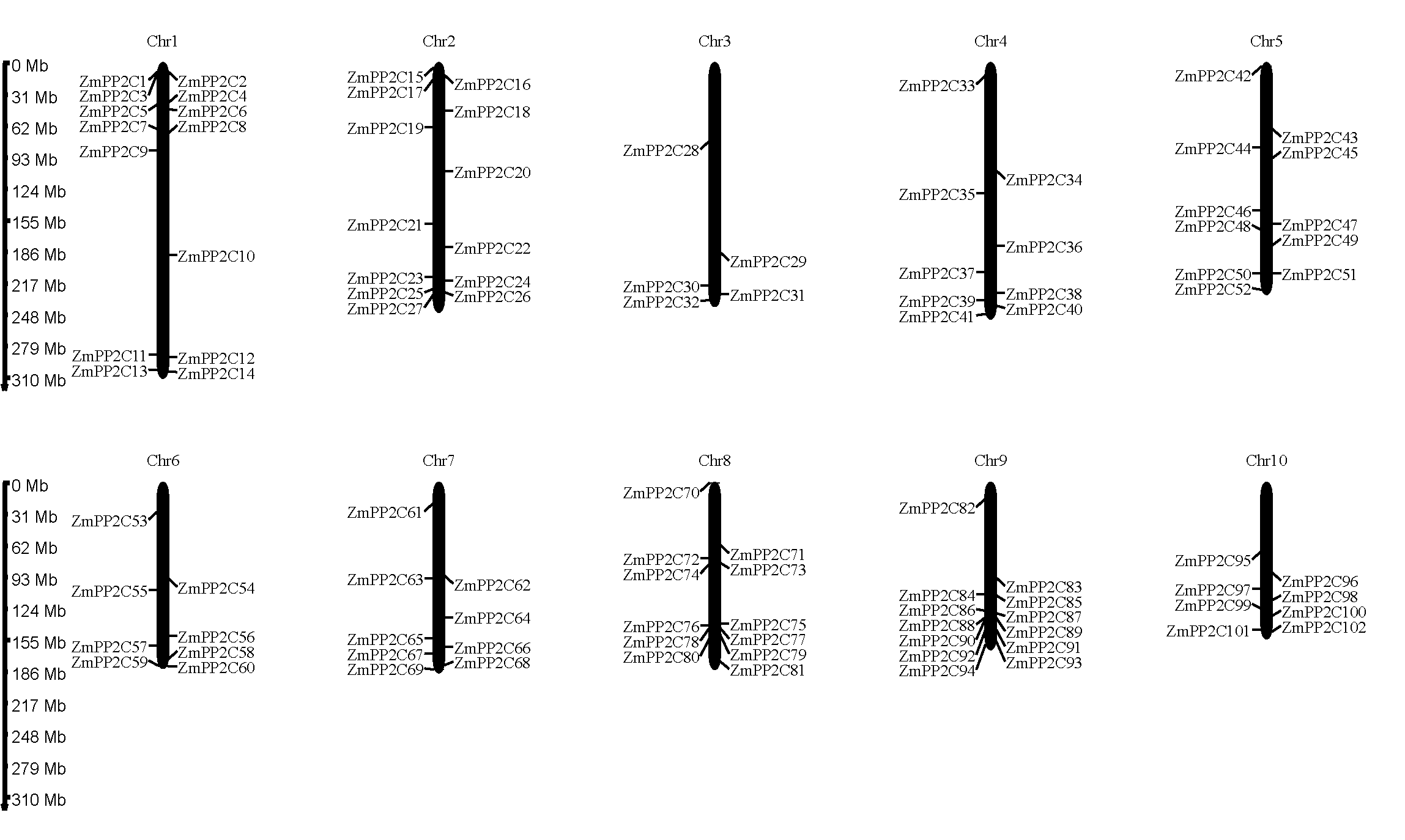

Supplement: Supplementary file 1 [file plants-13-00340-s001.zip › Supplementary Figure S1.tif]

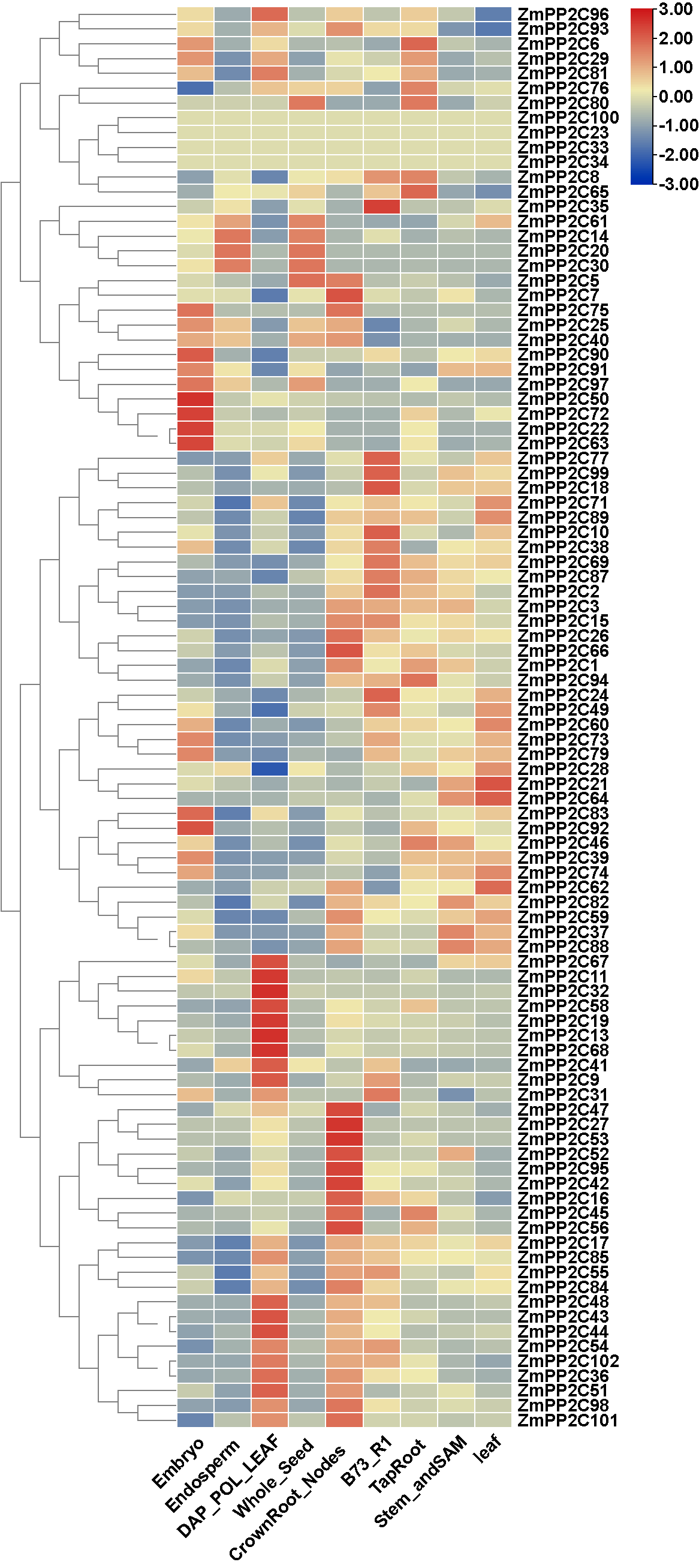

Supplement: Supplementary file 1 [file plants-13-00340-s001.zip › Supplementary Figure S2.tif]

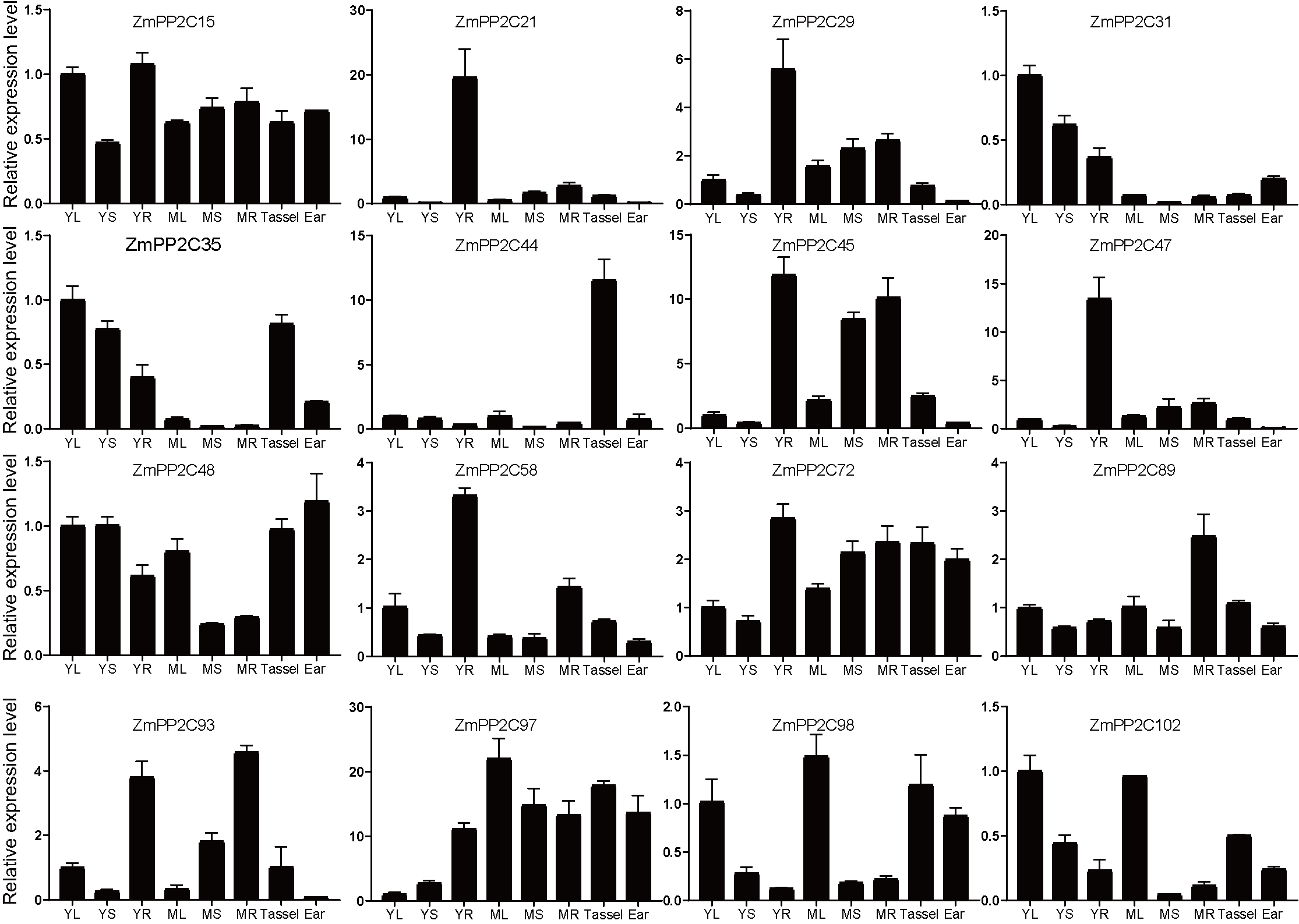

Supplement: Supplementary file 1 [file plants-13-00340-s001.zip › Supplementary Figure S3.tif]

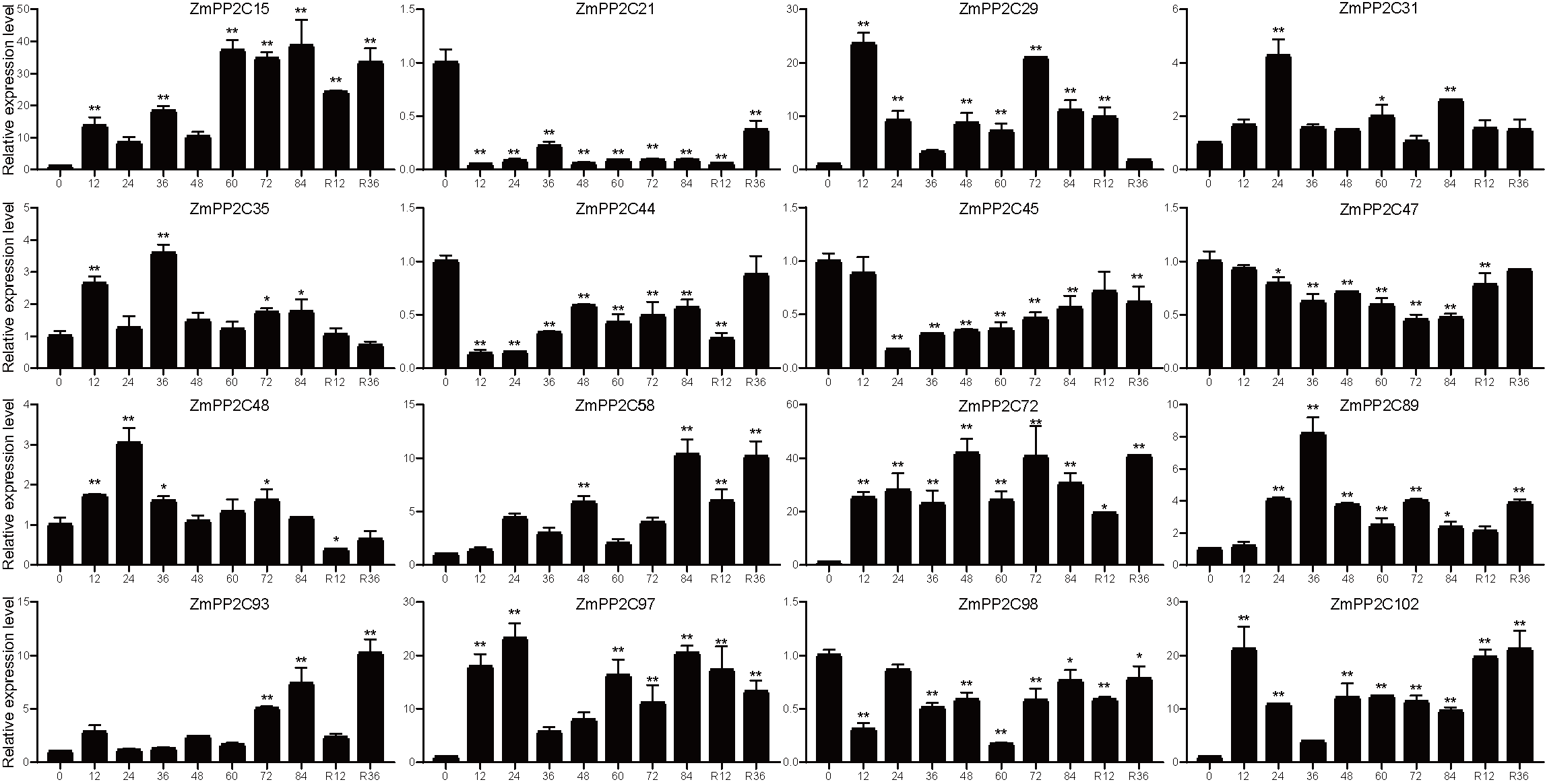

Supplement: Supplementary file 1 [file plants-13-00340-s001.zip › Supplementary Figure S4.tif]
